# ESRRG, ATP4A, and ATP4B as Diagnostic Biomarkers for Gastric Cancer: A Bioinformatic Analysis Based on Machine Learning

**DOI:** 10.3389/fphys.2022.905523

**Published:** 2022-06-23

**Authors:** Qiu Chen, Yu Wang, Yongjun Liu, Bin Xi

**Affiliations:** ^1^ Medical College, Yangzhou University, Yangzhou, China; ^2^ College of Physics Science and Technology, Yangzhou University, Yangzhou, China

**Keywords:** gastric cancer, machine learning, bioinformatics, WGCNA, diagnostic model

## Abstract

Based on multiple bioinformatics methods and machine learning techniques, this study was designed to explore potential hub genes of gastric cancer with a diagnostic value. The novel biomarkers were detected through multiple databases of gastric cancer–related genes. The NCBI Gene Expression Omnibus (GEO) database was used to obtain gene expression files. Three hub genes (*ESRRG*, *ATP4A*, and *ATP4B*) were detected through a combination of weighted gene co-expression network analysis (WGCNA), gene–gene interaction network analysis, and supervised feature selection method. GEPIA2 was used to verify the differences in the expression levels of the hub genes in normal and cancer tissues in the RNA-seq levels of Genotype-Tissue Expression (GTEx) and The Cancer Genome Atlas (TCGA) databases. The objectivity of potential hub genes was also verified by immunohistochemistry in the Human Protein Atlas (HPA) database and transcription factor–hub gene regulatory network. Machine learning (ML) methods including data pre-processing, model selection and cross-validation, and performance evaluation were examined on the hub-gene expression profiles in five Gene Expression Omnibus datasets and verified on a GEO external validation (EV) dataset. Six supervised learning models (support vector machine, random forest, k-nearest neighbors, neural network, decision tree, and eXtreme Gradient Boosting) and one semi-supervised learning model (label spreading) were established to evaluate the diagnostic value of biomarkers. Among the six supervised models, the support vector machine (SVM) algorithm was the most effective one according to calculated performance metrics, including 0.93 and 0.99 area under the curve (AUC) scores on the test and external validation datasets, respectively. Furthermore, the semi-supervised model could also successfully learn and predict sample types, achieving a 0.986 AUC score on the EV dataset, even when 10% samples in the five GEO datasets were labeled. In conclusion, three hub genes (*ATP4A*, *ATP4B*, and *ESRRG*) closely related to gastric cancer were mined, based on which the ML diagnostic model of gastric cancer was conducted.

## 1 Introduction

Gastric cancer (GC), reported as the sixth most common cancer in the world, has an extremely high morbidity rate (Sung et al., 2021). Latest global epidemiological data showed that almost 1,089,103 people were diagnosed with gastric cancer every year, and 768,793 people died of this disease, which makes it the fourth most fatal cancer worldwide (Sung et al., 2021). Although previous research studies have successfully revealed the major risk factors of GC, such as the genetic background, obesity, harmful mode of life, and *Helicobacter pylori* infection, a high rate of misdiagnosis still exists due to nonspecific symptoms at the beginning of the disease (Van Cutsem et al., 2016). In other words, GC usually has a late diagnosis at an advanced stage, resulting in its proximity to morbidity and mortality (Asplund et al., 2018). The prognosis of locally advanced gastric cancer is poor with a 5-year survival rate of 16.4% (Katai et al., 2018) and median overall survival (OS) of 6–14 months in East Asia after being diagnosed from extensive clinical studies (Hu et al., 2021). In contrast, if GC is diagnosed at an early stage, the 5-year survival rate is about 90% (Saragoni et al., 2013), indicating the importance of early diagnosis and treatment. Novel biomarkers screened through bioinformatics methods have already shown their potentiality in cancer development and diagnosis. Therefore, it is extremely meaningful to find novel biomarkers of GC to assist in the early diagnosis and treatment.

Recently, machine learning (ML) has been widely used as a bioinformatics method in the realm of medical data mining (Yang et al., 2020). Compared with traditional analyses, the ML technique has an edge on discovering hidden relationships and making predictions from complex datasets which have already been successful in many clinical practices, such as image-based cancer screening (Hu et al., 2018), constructing effective prognostic models (Royston et al., 2004), and identifying biomarkers based on the integration of omics and phenotype data (Subramanian et al., 2020). On the other side, biological networks such as weighted co-expression network analysis (WGCNA) (Langfelder and Horvath, 2008) and gene–gene interaction networks can identify the associations between genes and the biological processes. In accordance with biological network analyses, novel genes and pathways related to human cancers are also revealed (Boucher and Jenna, 2013; Farhadian et al., 2021). Thus, combing the core concepts of ML such as feature selection and classification with additional biological network analyses may further assist in exploring biomarkers with diagnostic values.

In this study, our purpose was to explore biomarkers based on biological network analyses and ML techniques, the novelty of which is further examined with ML diagnostic models. Potential hub genes are screened by the feature selection method and biological networks. ML diagnostic models are constructed by supervised and semi-supervised ML methods with stratified k-fold cross-validation and random permutation validation, respectively.

## 2 Materials and Methods

### 2.1 Data Collection and Preprocessing

**FIGURE 1:**
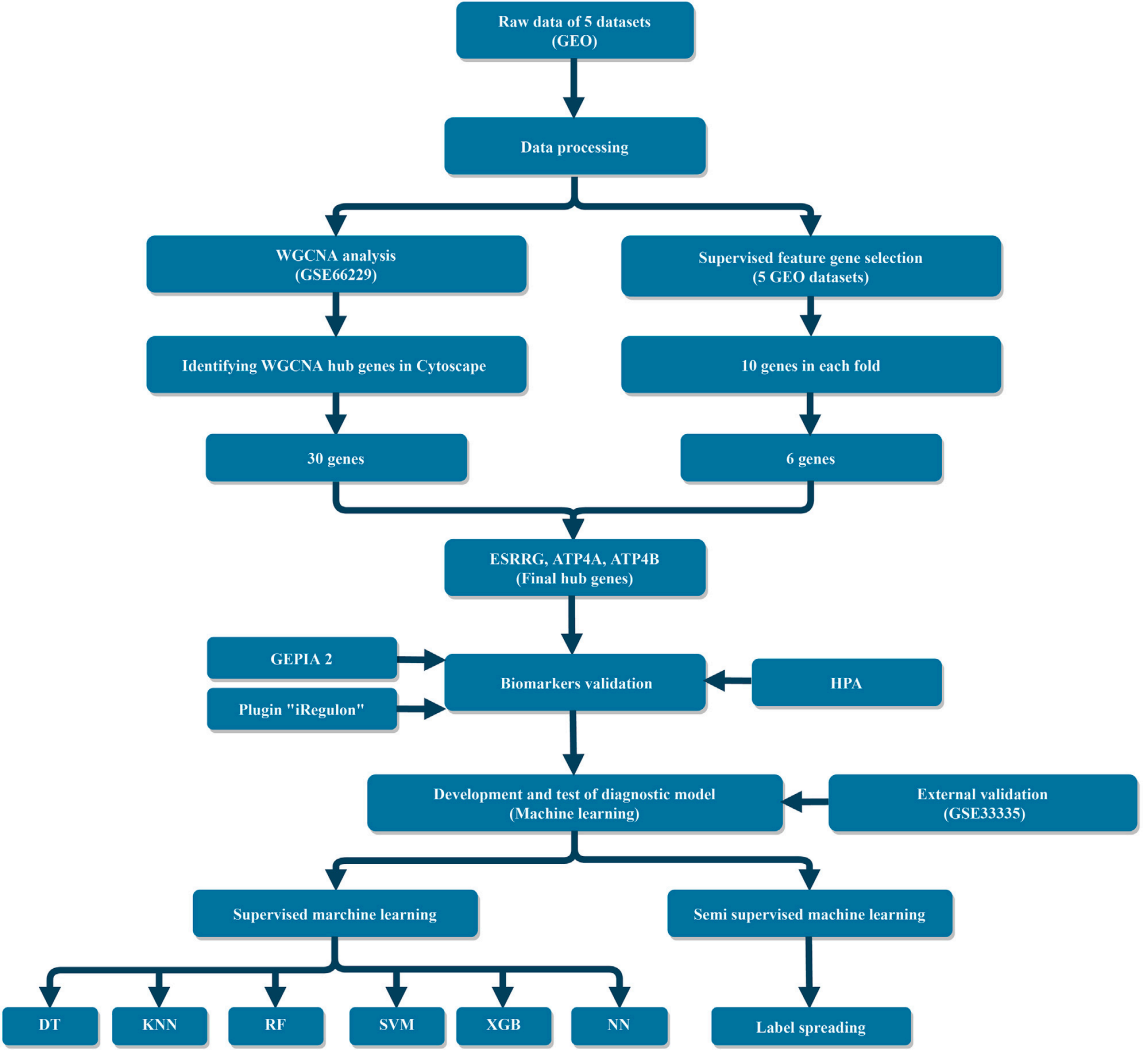
Flowchart of this study.

The study design is shown in Figure 1. This systematic study comprehensively downloaded six datasets from the Gene Expression Omnibus (GEO) database and focused on the gene sequencing results of GC patients with each dataset containing more than 10 samples. These datasets were produced using three different microarray platforms: Affymetrix Human Genome U133 Plus 2.0 Array, Affymetrix Human Exon 1.0 ST Array, and Affymetrix Human Genome U133A Array. Raw data of these datasets were preprocessed by R packages “oligo” (Carvalho and Irizarry, 2010) and “affy” (Gautier et al., 2004), and then, the background was corrected and normalized through the Robust Multichip Average (RMA) function. In this study, GSE66229 was used to construct a weighted gene co-expression network due to the sufficient data and detailed clinical characteristics of the gastric cancer samples. Five datasets (GSE19826, GSE27342, GSE29272, GSE54129, and GSE66229) were combined into a total dataset (TD) which contains 780 samples and 11,181 genes for feature selection and building ML models. TD includes 435 tumor samples and 345 normal ones, i.e., a mild imbalanced dataset. The combat algorithm in the “sva” R package (Johnson et al., 2007) was used to eliminate batch effects between different platforms and experiments. GSE33335 acts as an independent dataset, based on which an external validation (EV) was performed to validate the authenticity of hub genes and the reproducibility and generalizability of the ML diagnostic models. Details of all datasets can be found in Supplementary Table S1.

### 2.2 WGCNA

The R package “WGCNA” (Langfelder and Horvath, 2008) was constructed to detect gene modules, and the correlation of each module with sample type was evaluated. The specific steps are as follows: (a) in the GSE66229 dataset, only normal and cancer samples from the same individuals (196 samples) were selected for further analysis. Then, the 196 samples were divided into “tumor” and “normal” groups according to their clinical records, with each group containing 98 samples; (b) the samples were clustered by the “hclust” function to detect the outliers. After employing the “hclust” function to the expression matrix evaluated by the average method, 35 offending samples were removed with a height cut at 125; (c) the best scale-free topology fitting index (soft threshold) was selected as 7 to achieve a higher average network connectivity with a scale-free fitting number β=0.86 ; d) the adjacency matrix was transformed into a topological overlap matrix (TOM) to define the gene co-expression similarity; (e) Based on the dissimilarity measured by TOM, the “hclust” algorithm was employed for gene hierarchical clustering; (f) the optimal module size was set as 30, and the dynamic tree was used to cut the identification module; (g) after each module was determined based on the signature gene expression profile and the sample type of patients, the correlation of the module signature genes with sample types was also determined.

### 2.3 Identification of the WGCNA Hub Genes

Cytoscape (Shannon et al., 2003) was used to visualize the co-expression network in the modules of the highest correlations. All genes in the selected modules were exported to Cytoscape and analyzed with the “NetworkAnalyzer” plugin (Assenov et al., 2007), which can give a comprehensive set of topological parameters for gene–gene interaction networks. Hub genes are defined as genes with high connectivity in the gene–gene interaction network. According to connectivity, i.e., node degrees in the output of “NetworkAnalyzer”, the top-ranked 10% genes in the two most significant modules “red” and “turquoise” were selected (Fuxman Bass et al., 2013), which may have important implications for the progression of gastric cancer.

### 2.4 Supervised Feature Gene Selection With the Fisher Score Algorithm

The feature selection technique is a process of reducing the number of variables, especially important for developing a predictive model (Ali et al., 2018). The feature selection method can evaluate the relationship between each variable and the output and select those variables with the strongest relationship. Fisher score is one of the most widely used supervised feature selection methods, which returns the ranks of the variables based on the Fisher score in the descending order (Gu et al., 2011). The Fisher score $S_{i}$ of the i -th feature is calculated as follows:

$$\begin{array}{c} S_{i}=\frac{\sum_{j}n_{j}{\left(\mu_{ij}-\mu_{i}\right)}^{2}\;\;}{\sum_{j}n_{j}\sigma_{ij}^{2}}, \end{array}$$ (1)

where $\mu_{ij}$ and $\sigma_{ij}$ are the mean and standard deviation of the i -th feature in the j -th class, respectively. $n_{j}$ is the number of samples in the j -th class, and $\mu_{i}$ is the mean of the i -th feature.

In this study, to select the most relevant genes that are strongly related to the sample type, feature selection using the Fisher score algorithm was applied to the combined five datasets. Here, a gene was regarded as a feature, and TD was splitted into five folds during feature selection. A list of genes ranked by their scores returned in each fold, where we picked the top-ranked 10 feature genes with a cutoff at $S_{i}\approx 0.5$ for each list for further study. The feature genes were determined as the intersection of the features of five folds. The final biomarkers in this study were obtained by the intersection of the hub genes filtered by the gene–gene interaction network and the feature genes.

### 2.5 Validation of the Final Hub Genes

GEPIA2 can be used to verify the expression difference of the hub genes in tumor samples and normal ones (Tang et al., 2019). The RNA-seq datasets used in GEPIA2 were based on UCSC Xena (http://xena.ucsc.edu), which was computed by standard pipelines to analyze the RNA-sequencing expression of tumor and normal samples from the TCGA (Colaprico et al., 2016) and GTEx (Lonsdale et al., 2013) datasets. In this study, we used the TCGA and GTEx gastric cancer RNA-seq data integrated by the GEPIA2 platform for a comprehensive validation. With |Log_2_FC| cutoff = 1 and *p*-value cutoff = 0.01, box plots of the RNA-seq data of the gastric cancer hub genes were drawn.

The immunohistochemistry (IHC) staining data for this study were downloaded from the Human Protein Atlas (HPA) database (Thul and Lindskog, 2018), and then, the results of gastric cancer pathology and normal gastric tissue were processed.

The Cytoscape plugin “iRegulon” was used to analyze the transcription factors regulating hub genes (Janky et al., 2014; Gao et al., 2020). This plugin predicts transcription factors by using the motif enrichment analysis and using track discovery in a set of regulated genes. The cutoff criteria were as follows: enrichment score threshold = 3.0, receiver operating characteristic (ROC) threshold for area under the curve (AUC) calculation = 0.03, rank threshold = 5,000, minimum identity between orthologous genes = 0.0, and false discovery rate (FDR) = 0.001. After all transcription factors were outputted, the factor which regulates all hub genes and ranks first in the normalized enrichment score (NES) was defined as the most relevant transcription factor.

### 2.6 Development and Validation of Machine Learning Models

#### 2.6.1 Supervised Learning

At the first step, TD was randomly split into training and test datasets, with ratios of 80 and 20%, respectively. Then, a repeated stratified k-fold cross-validation was performed on the training dataset. The stratified k-fold can ensure that each fold has the same proportion of the sample type compared to the whole one, which is more suitable to imbalance datasets. The ML model was trained using of k-1 folds and validated on the one remaining fold for k times. The training performance of the model was reported on the average over k times. At last, a final evaluation was performed on the test dataset. The aforementioned steps can be regarded as an internal validation since both training and test datasets come from TD. To examine the robustness of the ML models, an EV was further performed on the independent dataset GSE33335.

In this study, k was set to 10, and the cross-validation was repeated 100 times with different randomizations in each repetition to ensure the estimated performance. The Matthews correlation coefficient (MCC) metric (Chicco and Jurman, 2020) was chosen as the performance score for the model evaluation during the training process, which is suitable to imbalance datasets.

To further reduce the affection of the dataset imbalance, the synthetic minority oversampling technique (SMOTE) was applied to the training dataset (Chawla et al., 2002). The SMOTE can synthesize new samples based on randomly picked existing samples and their k-nearest neighbors. In this study, a grid search of k ranging from 1 to 7 was also performed.

In order to select a proper classifier for the ML diagnosis model, six widely used algorithms, namely, support vector machine (SVM) (Byvatov and Schneider, 2003), k-nearest neighbors (KNN) (Zhang, 2016), decision tree (DT) (Chen et al., 2011), random forest (RF) (Chen and Ishwaran, 2012), neural network (NN) (Lancashire et al., 2009), and eXtreme Gradient Boosting (XGB) (Chen and Guestrin, 2016), were examined through their performance metrics for classification results. Hyperparameters of all the models were finely tuned using the scikit-learn GridSearchCV method, according to the highest “MCC” scores. The best model for each algorithm was selected after exploration of the whole grid. Best hyperparameters and the corresponding training performances of all supervised ML diagnostic models can be found in Table 1. Finally, the performance of each model on test and EV datasets was evaluated by these performance metrics: accuracy (Heidaryan, 2018), specificity (Altman and Bland, 1994), sensitivity (Altman and Bland, 1994), precision (Heidaryan, 2018), F1 score (Chicco and Jurman, 2020), and MCC. Furthermore, the ROC curve and AUC are also given.

**TABLE 1:** All genes and their Fisher Scores were selected by the feature algorithm.

Fold 1		Fold 2		Fold 3		Fold 4		Fold 5	
Gene name	Fisher score	Gene name	Fisher score	Gene name	Fisher score	Gene name	Fisher score	Gene name	Fisher score
ATP4A	0.762	ATP4A	0.745	ATP4A	0.796	ATP4A	0.862	ATP4A	0.810
ESRRG	0.736	ESRRG	0.705	ESRRG	0.734	ESRRG	0.803	ESRRG	0.749
CBLIF	0.671	ATP4B	0.642	CBLIF	0.642	CBLIF	0.748	CBLIF	0.670
ATP4B	0.641	CBLIF	0.632	ATP4B	0.631	ATP4B	0.712	ATP4B	0.644
INHBA	0.548	TIMP1	0.574	SST	0.540	INHBA	0.618	KCNE2	0.553
KCNE2	0.541	KCNE2	0.517	MT1M	0.539	KCNE2	0.615	TIMP1	0.541
CPA2	0.533	INHBA	0.513	TIMP1	0.538	CPA2	0.601	INHBA	0.524
MT1M	0.531	CPA2	0.498	INHBA	0.522	TIMP1	0.595	CPA2	0.520
ALDH6A1	0.529	MT1M	0.491	KCNE2	0.511	MYRIP	0.587	MYRIP	0.505
TIMP1	0.510	GKN1	0.468	GKN1	0.501	MT1M	0.543	MT1M	0.494

#### 2.6.2 Semi-Supervised Learning

To deal with a different problem, such as handling large amounts of samples with only a few diagnosed ones, a semi-supervised learning model based on a label-spreading algorithm is also examined (Zhou et al., 2003). Semi-supervised learning can learn from small amounts of labeled samples, combined with the use of unlabeled data to better capture the underlying properties and generalize better to new samples (Chapelle et al., 2009). To some extent, semi-supervised learning can be regarded as a hybrid of supervised and unsupervised learning. In this study, TD was randomly split into a labeled dataset and an unlabeled one, with five unlabeled ratios including 50, 60, 70, 80, and 90%. For each ratio, the semi-supervised model was cross-validated by 100 times of random permutations and further evaluated on the EV dataset GSE33335. The performance metrics of prediction on the unlabeled dataset and EV dataset are given.

All supervised and semi-supervised ML models in this study were implemented by Python language programming on Intel Xeon silver 4110 CPU.

## 3 Results

### 3.1 Construction of the Gene Co-Expression Network

**FIGURE 2:**
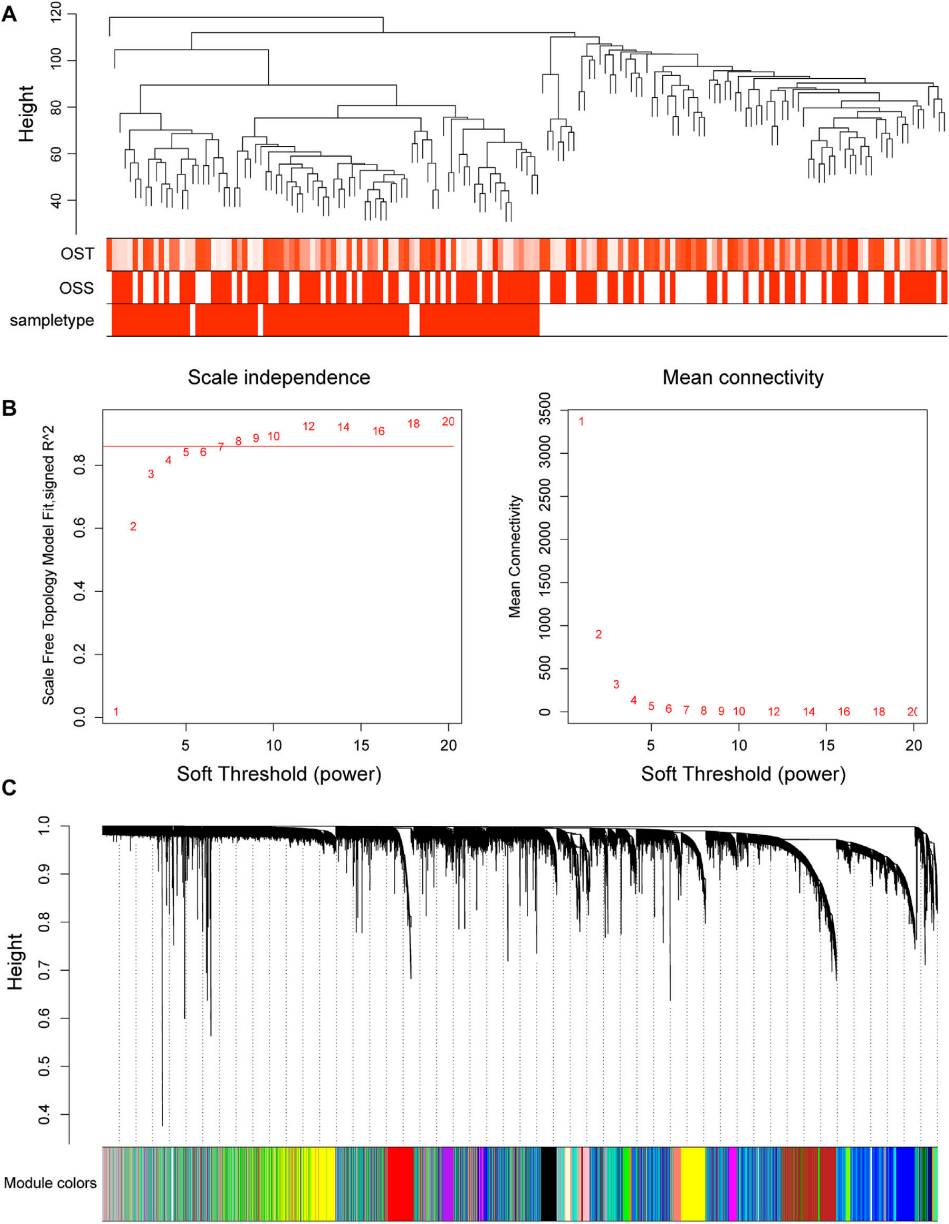
Progress of the weighted gene co-expression network analysis in GSE66229. **(A)** Cluster dendrogram of 161 samples in GSE66229. **(B)** Soft thresholds of the best scale-free topological model fitting index (left) and mean connectivity (right) were determined. The red horizontal line represents *R* ^2^ = 0.86. **(C)** Dendrogram of all genes clustered in GSE66229. Gene clustering into modules is based on a topological overlap matrix. Assigned modules are colored on the bottom with gray denoting unassigned genes.

**FIGURE 3:**
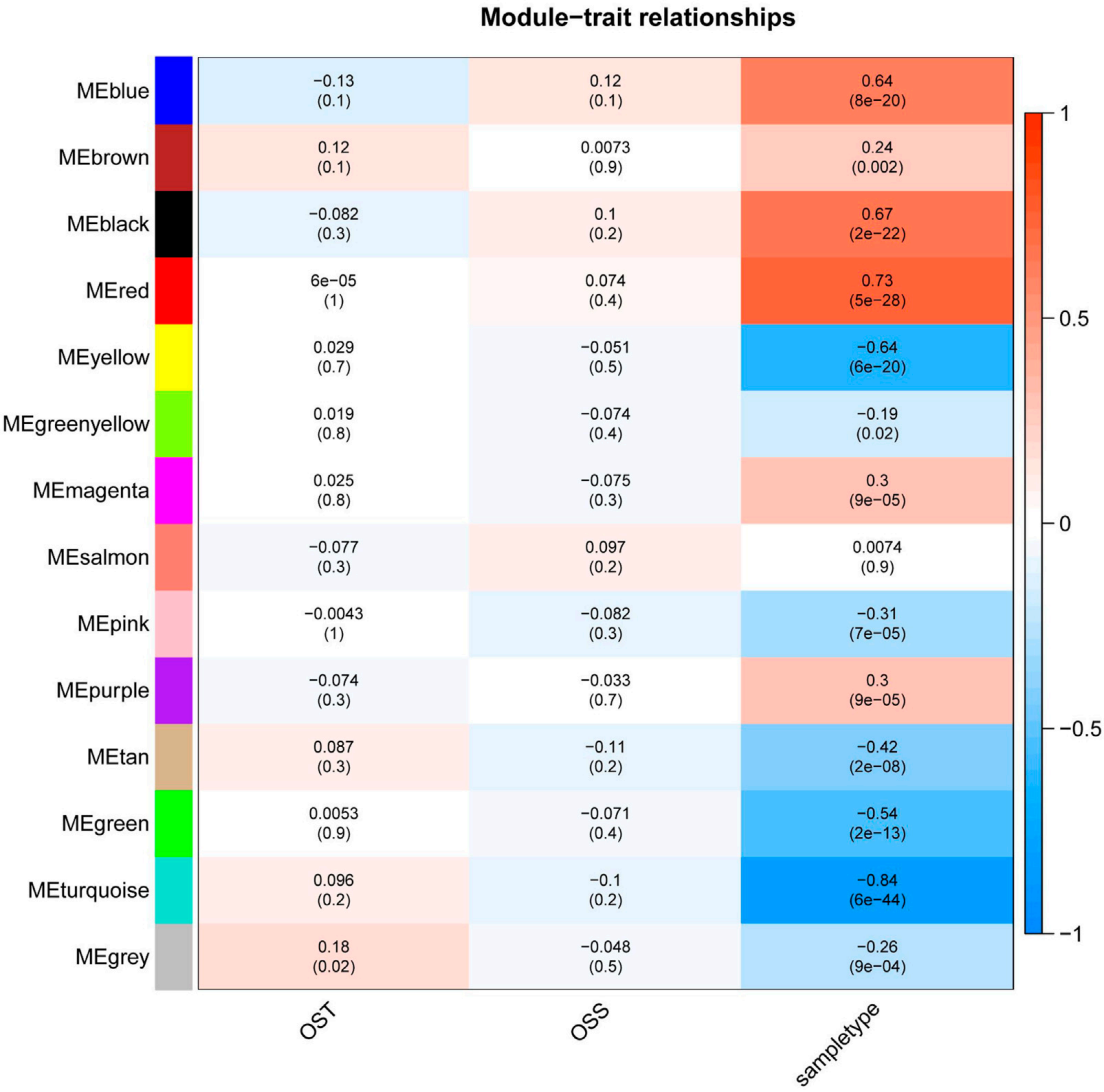
Heatmap of the relationship between module eigengenes and clinical traits of GSE66229. WGCNA labeled heatmaps for GSE66229, each row represents a module characteristic gene encoded by color, and the three columns represent clinical characteristics of overall survival time (OST), overall survival status (OSS), and sample type, respectively. Each cell represents the Pearson correlation coefficient and *p*-value (in parentheses) of the corresponding module characteristics, and the color of each cell represents the value of correlation.

In order to find the correlation between clinical features and genes, this study used the R “WGCNA” package to construct 20,862 genes and 161 samples in the GSE66229 dataset into a gene network. A sample clustering figure was plotted (Figure 2A). To guarantee a scale-free topology and zero mean connectivity, the threshold was determined to be 7 (Figure 2B). The dissimilarity of the modules was set as 0.2, and a total of 14 modules were generated (Figure 2C). Two modules (red: r = 0.73 and P = 5e-28; turquoise: r = -0.84 and P = 6e-44) with the positive and negative highest correlations were acquired as the significant modules for subsequent analyses (Figure 3).

### 3.2 Feature Gene Selection

In order to select critical genes to the diagnostic model, feature selection was performed for the combined five datasets using the Fisher Score method on five folds. In each fold, a cutoff around the Fisher Score $S_{i}\approx 0.5$ s was applied, and as a result, 10 genes with the highest scores were selected. All selected genes as well as their Fisher Scores are listed in Table 1. At last, the intersection of all picked genes in the five folds is investigated, resulting in six intersection elements: TIMP1, ATP4A, ESRRG, CBLIF, ATP4B, and INHB.

### 3.3 Identification and Validation of Hub Genes

**FIGURE 4:**
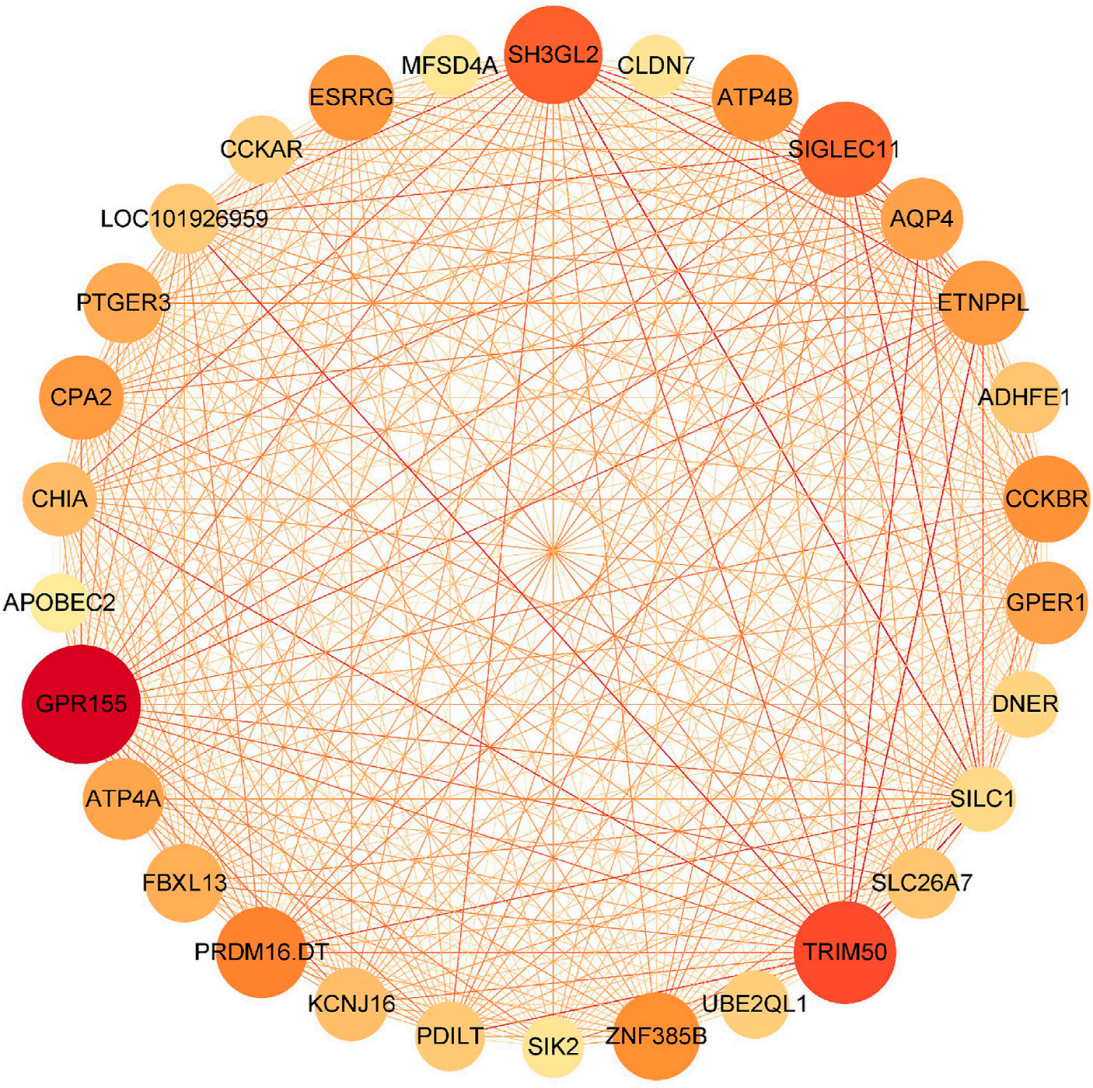
Gene–gene interaction network of the top-ranked 10% genes in red modules.

In the results of WGCNA, two significant models, the red and turquoise ones, were exported to Cytoscape. Two gene–gene interaction networks were constructed and analyzed in Cytoscape. Then, the top 10% target genes of each network were selected, according to the connectivity degree. As a result, 30 and 330 genes in the “red” and “turquoise” modules were selected. The gene–gene network of 30 genes in the “red” module is shown in (Figure 4), while genes in the “turquoise” module are listed in Supplementary material S1. Together with the six feature genes selected through the Fisher Score method, three hub genes (*ESRRG*, *ATP4A*, and *ATP4B*) were finally selected in the red module, while none was selected in the turquoise module.

**FIGURE 5:**
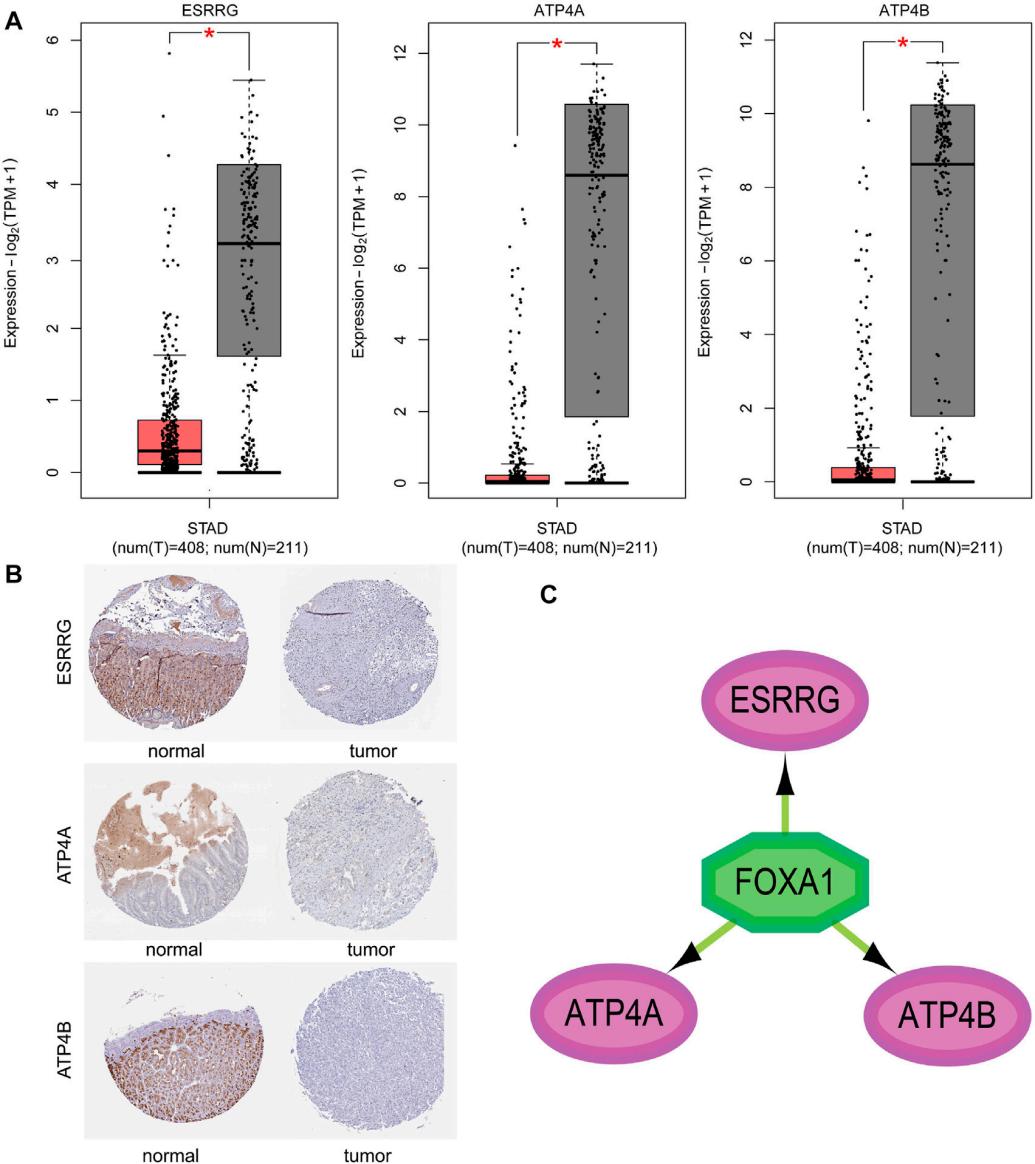
Validation of three hub gene expressions in the GEPIA2 platform. **(A)** Validation of three hub gene expressions in the GEPIA2 platform. The red and gray boxes represent cancer and normal tissues in the TCGA and GTEx datasets, respectively. STAD, gastic cancer, and *p* < 0.01 (GEPIA2 website). **(B)** Immunohistochemical staining of ESRRG, ATP4A, and ATP4B in the Human Protein Atlas (HPA) database. **(C)** Transcription factor–hub gene regulatory network of the most relevant factor in the Cytoscape plugin “iRegulon”.

The expression of three genes in cancer and normal samples was validated in GEPIA2. The box plot of GEPIA2 presented the expression levels of the three genes in the standard of expression-log2 (TPM+1) (Figure 5A). We observed that the expressions of ESSRG, ATP4A, and ATP4B in tumor samples were significantly lower than those in normal ones. This study also performed an IHC analysis in the gastric data of hub genes from the HPA database. The results of IHC staining are shown in Figure 5B, which were consistent with GEPIA2.

Among all transcription factors regulating the three hub genes, FOXA1 with the highest NES (NES = 5.142) was considered as the most important transcription factors (Figure 5C). Existing studies have shown that the expression of FOXA1 affects the proliferation and invasion of gastric cancer cells (Lin et al., 2018; Dai Y. et al., 2021). The result verified the objectivity of the three hub genes in gastric cancer.

### 3.4 Establishment and Validation of the Machine Learning Model

**FIGURE 6:**
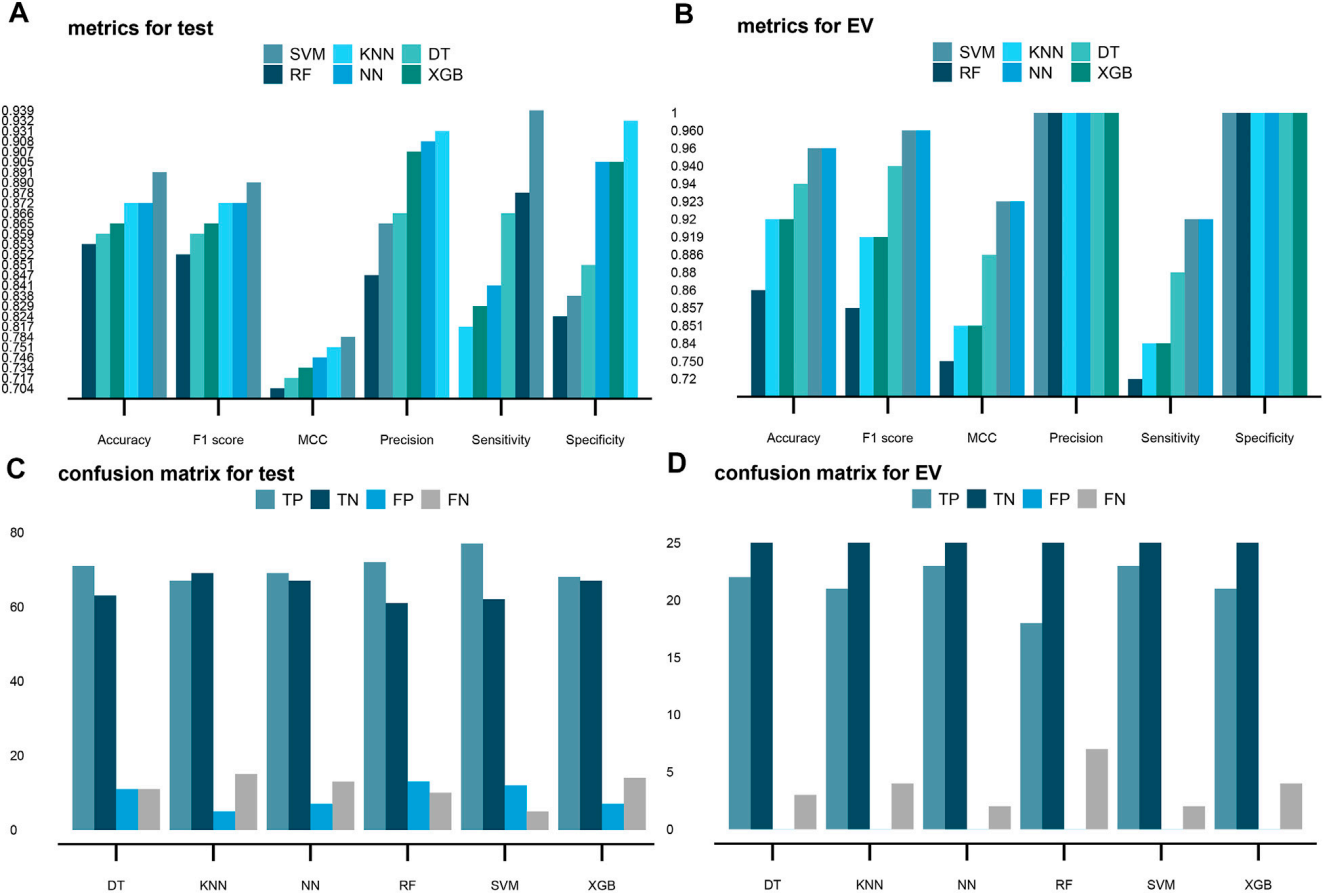
Performance of the six supervised machine learning models on the test and EV sets. Hyperparameters of all six models are tuned with the GridSearchCV method, according to the “MCC” metric, and then, the six best models were chosen after exploration of the whole grid. Predictions on the test and EV sets are made with the best models. Six models used in this study are support vector machine (SVM), k-nearest neighbors (KNN), decision tree (DT), random forest (RF), neural network (NN), and eXtreme Gradient Boosting (XGB) in order. **(A,B)** Scores of accuracy, F1 score, MCC, precision, sensitivity, and specificity in the six models on the test and valid datasets, respectively. **(C,D)** Four terms of the confusion matrix (TP, TN, FP, and FN) in the six models on the test and valid datasets, respectively.

After going through the hyperparameter grid and the SMOTE grid, the best model was selected according to the MCC metric. The corresponding hyperparameters, k-nearest neighbors in the SMOTE, and values of MCC on the training dataset are listed in Table 2. One might see that the SVM model has the best performance with an average MCC score at 0.666±0.093 .With the fixed hyperparameters, the performance of all six ML diagnostic models on the test dataset is shown in Figure 6. The trained SVM had the highest accuracy with 89.1%, while the RF showed the lowest but a close accuracy with 85.3% (Figure 6A), which demonstrated the robustness of both hub genes and ML methods.

**TABLE 2:** Tuned hyperparameters, k in the SMOTE, and the training performance of six machine learning models.

Model	Tuned hyperparameters	k	MCC
SVM	C: 3,000 and gamma: 0.01	5	0.666 ± 0.093
RF	max_features: log2 and n_estimators: 1,500	5	0.641 ± 0.091
KNN	metric: manhattan and n_neighbors: 29	3	0.649 ± 0.089
NN	activation: tanh and hidden_layer_sizes: (200, 200, and 200)	6	0.633 ± 0.096
DT	max_depth: 60, min_impurity_decrease: 0.2, and min_samples_leaf: 2	7	0.637 ± 0.090
XGB	gamma: 1, max_depth: 2, and n_estimators: 100	5	0.658 ± 0.092

Based on the results, the weakest performance of the sensitivity metric was the KNN algorithm with a ratio of 81.7% (Figure 6A). As a contrast, the SVM algorithm again had the highest sensitivity of 93.9%, showing the great ability for predicting tumor samples (Figure 6A). For specificity, the NN algorithm had the best performance with a 90.5% specificity to predict normal samples. The RF algorithm had the lowest specificity of 82.4%. The SVM algorithm had the second lowest specificity of 83.8% (Figure 6A). These results demonstrated the six models have both advantages and disadvantages.

MCC and F1 scores could serve as more reliable metrics which involve all four terms: true positive (TP), true negative (TN), false positive (FP), and false negative (FN) in the confusion matrix. According to the ratios of the MCC and F1 scores, the SVM should be the best model with 78.4 and 89%, respectively (Figure 6A).

**FIGURE 7:**
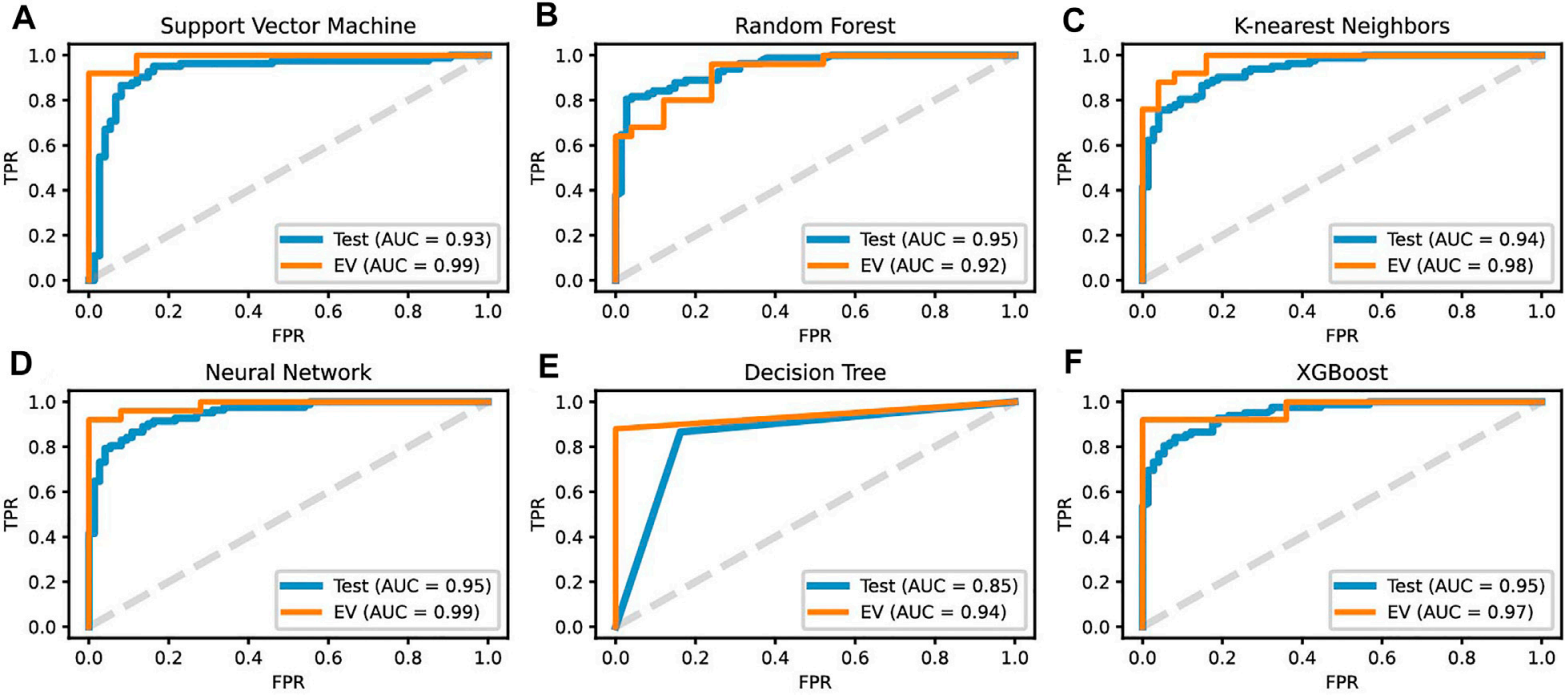
ROC curves for the predicted probability on the test and EV sets of all six machine learning diagnostic models: **(A)** SVM, **(B)** RF, **(C)** KNN, **(D)** NN, **(E)** DT and **(F)** XGB.

ROC curves for all six ML classification diagnostic assistants were built using the predicted probability of belonging to different classes. Except for the AUC of DT having the lowest value of 85%, all the other models had an AUC of 93–95% (Figure 7).

**FIGURE 8:**
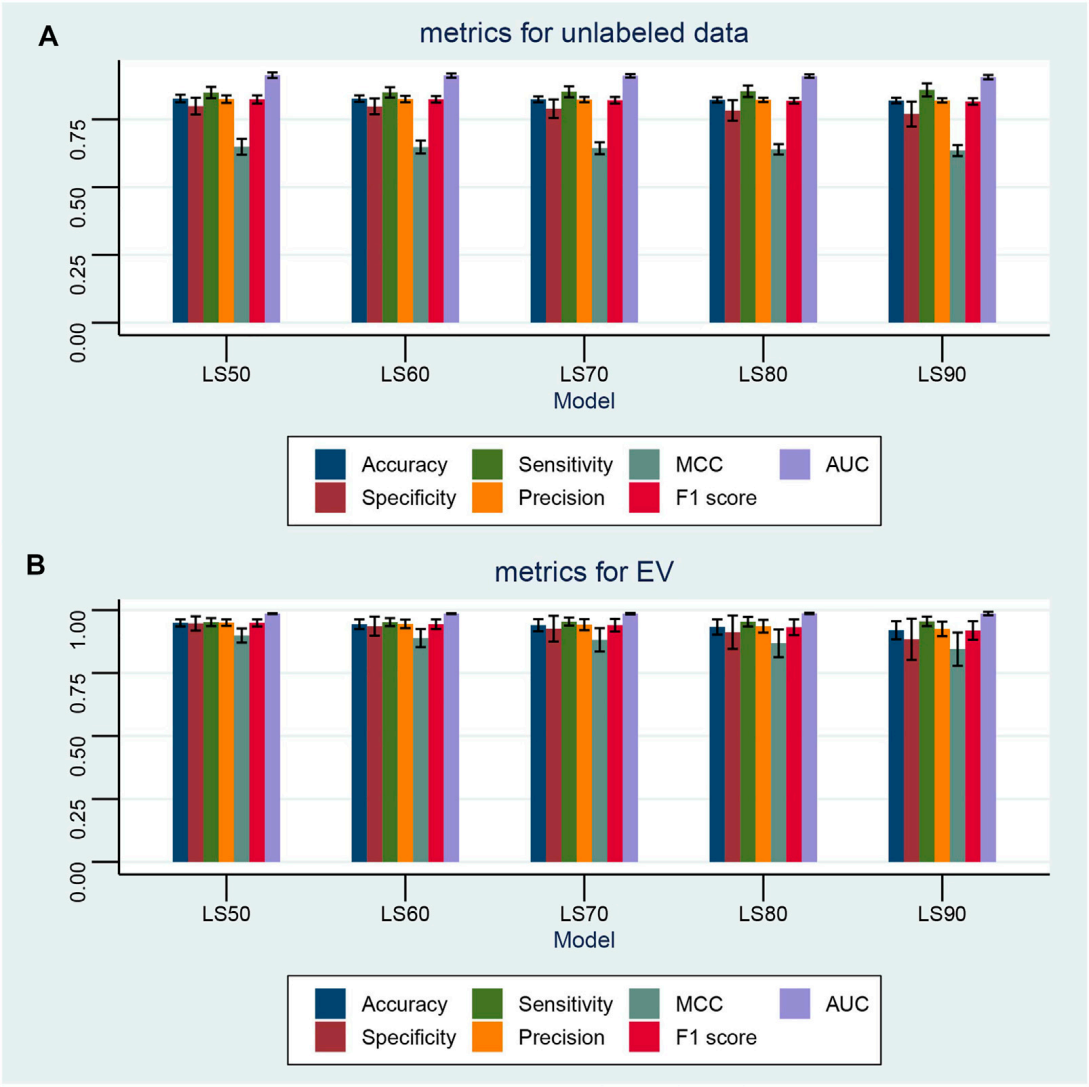
Performance of the semi-supervised machine learning model with various ratios of unlabeled data. Semi-supervised machine learning models are built with the label spreading (LS) algorithm. The ratios of randomly unlabeled samples include 50% (LS50), 60% (LS60), 70% (LS70), 80% (LS80), and 90% (LS90). In each ratio, the semi-supervised model is cross-validated 100 times by random permutation. **(A,B)** Performance of the semi-supervised machine learning models on all unlabeled data and the valid dataset with various ratios of unknown samples, respectively. Seven metrics are given, namely, accuracy, F1 score, MCC, precision, sensitivity, specificity, and AUC.

The prediction performance of six ML diagnostic assistants was further evaluated on the EV dataset (25 tumor samples and 25 normal ones). The results showed the six models can classify all the normal samples correctly with specificity and precision both equaling to 1 (Figure 8B); however, the prediction on tumor samples varies. SVM and NN have the best performances on successively predicting 23 tumor samples (Figure 6D). As a result, SVM and NN share the highest MCC and F1 scores of 92.3 and 96%, respectively (Figure 6B). The AUC of SVM and NN on EV is 99% (Figures 7A, D). Therefore, one can conclude that the SVM model based on the expression profiles of three hub genes may have a potential diagnostic value for gastric cancer.

### 3.5 Semi-Supervised Diagnostic Model

Semi-supervised ML can learn from a combination of small amounts of labeled samples and large amounts of unlabeled ones, which is especially suitable for the scenario of annotating large amounts of samples with expensive costs or miscellaneous steps. In this study, the label spreading (LS) algorithm was tested on 50% (LS50), 60% (LS60), 70% (LS70), 80% (LS80), and 90% (LS90) randomly unlabeled samples in TD. Each learning model was cross-validated 100 times with random permutation. The results shown in Figure 8 demonstrate that the LS algorithm can successfully learn and predict the sample type even when small amounts of labeled data are available. The mean MCC and F1 scores are 0.649 ± 0.029 and 0.824 ± 0.015, respectively, with 50% unlabeled samples. As the ratio of unlabeled samples increases, the performance of the LS slightly decreases. However, with 90% unlabeled data, the LS90 model still has mean MCC and F1 scores of 0.635 ± 0.020 and 0.816 ± 0.012, respectively. Furthermore, all LS models achieved a good prediction performance for the EV dataset, for example, the LS90 model has mean MCC and F1 scores of 0.845 ± 0.066 and 0.919 ± 0.037, respectively.

## 4 Discussion and Conclusion

Gastric cancer is still a major disease threatening human health, so it is particularly important to find a comprehensive and effective set of biomarkers with diagnostic values. This study systematically used a series of bioinformatics methods to select key features, i.e., hub genes, which were further confirmed by both, the GEPIA2 tool and IHC experiments. The transcription factor–hub gene regulatory network confirmed that three genes are closely associated with gastric cancer in the level of transcription factors. Based on these features, ML diagnostic assistants for the diagnosis of gastric cancer were established by both supervised and semi-supervised learning. The performance of the ML models on the EV dataset further approves the potential diagnostic ability.

In this study, five GEO datasets were downloaded for construction, and one independent GEO dataset GSE33335 was used for external validation. Comprehensive data collation can make the construction of diagnostic assistants more objective (Ahluwalia et al., 2021; Dai W. et al., 2021; Ye et al., 2021). GSE66229 was used for the WGCNA analysis. WGCNA is a widely used target therapy analysis tool, which clusters related genes, according to some clinical characteristics of research subjects. There have been many studies on gastric cancer tumor markers in recent years, and most of the WGCNA clusterings are based on differentially expressed genes (DEGs) in the research dataset (Li et al., 2021; Xiang et al., 2021; Zhang et al., 2022). In contrast, this study performed the WGCNA analysis on all gastric cancer–related genes in one dataset and fused them with selected features using a supervised learning method, i.e., Fisher score algorithm on five combined datasets, preserving the diversity of the gastric cancer hub genes. This also reduces the hub gene bias caused by clustering with a certain clinical feature traditionally (Yang et al., 2022). We put WGCNA-significant modules into Cytoscape to construct a gene–gene interaction network. Previous research shows that gene–gene interaction networks can reveal the principle and mechanisms of cancer (Zeng et al., 2013; Rana et al., 2020). In order to enhance the objectivity and authenticity of the hub genes, genes that are highly associated with gastric cancer screened by the gene–gene interaction network were intersected with the selected feature genes.

Three hub genes were crucial to the next machine learning-based bioinformatics approach. Although no studies used them as combined biomarkers for gastric cancer diagnosis, some studies have screened these genes in the identification of gastric cancer biomarkers and explored them to a certain extent in the field of human and animal experiments on gastric cancer (Lozano-Pope et al., 2017; Peng et al., 2020; Liu et al., 2021). ESRRG belongs to the estrogen-related receptor family. In one aspect, it has been classified that ESRRG inhibits the occurrence of gastric cancer by inhibiting the Wnt pathway by activating DY131 (Kang et al., 2018). In another, ESRRG can directly bind to the TFF1 promoter, which is a recognized tumor suppressor and inhibits *Helicobacter pylori* infection (Kang et al., 2021). *Helicobacter pylori* infection is a common cause of chronic atrophic gastritis, which is a precancerous lesion (Rolig et al., 2012). ATP4A and ATP4B belong to a family of P-type cation-transporting ATPases. These two genes belong to the gastric proton pump and are antigens of gastric parietal cells, which are diagnostic markers for immune gastric lesions including atrophic gastritis. Through *in vivo* and *in vitro* experiments in animals and humans, researchers have found that ATP4A and ATP4B were partially or fully methylated in gastric cancer cells. It was also verified that the reactivation and demethylation of ATP4A and ATP4B can effectively inhibit the progression of gastric cancer (Lin et al., 2017; Cao et al., 2020). Hence, ATP4A and ATP4B are important tumor suppressor genes.

Six supervised diagnostic models and one semi-supervised diagnostic model were developed based on different algorithms including SVM, RF, KNN, DT, NN, and XGB (supervised), and LS (semi-supervised). The performance was evaluated by seven metrics, namely, accuracy, specificity, sensitivity, precision, MCC, F1 score, and AUC. All the models were trained through cross-validation and further examined on the EV dataset GSE33335. The results suggested that SVM and LS can serve as the most appropriate algorithm for prediction. For example, LS90 can learn from only 10% of labeled data and achieve 0.906 ± 0.008 and 0.986 ± 0.007 AUC scores for 90% unlabeled data and the EV dataset. Therefore, this study demonstrates the potential ability of the ML diagnostic model created with three hub gene expression profiles of 780 samples.

In recent years, bioinformatics analyses based on machine learning have been popularly used in individual medicine. For example, multi-classifiers and deep neural networks are being applied in cancer research (Huang et al., 2018; Zhang et al., 2021). Comparing to previous studies, our research may be more robust in model development and evaluation. First, we included five datasets with 780 samples in the model development and internal validation. Second, we also used an independent dataset only for external validation. Huang et al. (2018) applied multi-classifiers to select gastric cancer-related miRNAs in one dataset and validate their performance in another two datasets. Huang *et al.* and our team both explored the application of SVM in the diagnosis of gastric cancer. Their SVM diagnostic model’s AUC was 95% in the training dataset, which is slightly higher than our corresponding AUC (93%). However, their model achieved a biased performance on the two valid datasets: one was 97%, while the other was less than 80%. Relatively fewer samples in model development may be responsible for this performance. Moreover, their two validation datasets were also involved in biomarker selection; thus, they might not be totally independent. Compared with the WGCNA and network control analyses used in our study to screen potential cancer-related genes, Zhang et al. (2021) fused gene expression data and DNA methylation data to obtain relatively more biomarkers for training their deep neural networks. On one hand, their study got an extremely high performance in six metrics. The accuracy, precision, recall, F1 score, and AUC value were all around 99%. On the other hand, the absence of an external validation report makes the generalization ability of their study remain unclear.

More several strengths of this study should be emphasized. First of all, data sources in this study come from Asia. Consistency in data sources may strengthen the pertinence of the model. Second, rich data in six datasets are sorted and then integrated into a comprehensive one to build an objective and effective diagnostic model. Third, hub genes selected from three robust methods were used in combination (WGCNA, gene–gene interaction network, and feature gene selection). Fourth, the selected hub genes are multiple-validated by GEPIA2, HPA databases, and transcription factor–hub gene regulatory network, the results of which further confirm the importance of the selected biomarkers. Finally, the diagnostic model is improved with the SMOTE and passes advanced machine learning analysis on an EV dataset and presented more convincing statistical results than previous studies. This study still has some flaws. First, this study deserves to be verified by subsequent independent experiments. Second, although comprehensive bioinformatics analyses were conducted in this study, an in-depth mechanistic study of three hub genes had not been advanced.

Finally, this study systematically established a gastric cancer diagnostic assistant based on multi-database bioinformatics and machine learning analysis. Our results have a moderate effect on auxiliary diagnosis. We expect future research to test the stability of the model.

## Data Availability

The datasets presented in this study can be found in online repositories. The names of the repository/repositories and accession number(s) can be found in the article/Supplementary Material.

## References

[B1] Ahluwalia P. Kolhe R. Gahlay G. K. (2021). The Clinical Relevance of Gene Expression Based Prognostic Signatures in Colorectal Cancer. Biochimica Biophysica Acta (BBA) - Rev. Cancer 1875 (2), 188513. 10.1016/j.bbcan.2021.188513 33493614

[B2] Ali H. E. A. Lung P.-Y. Sholl A. B. Gad S. A. Bustamante J. J. Ali H. I. (2018). Dysregulated Gene Expression Predicts Tumor Aggressiveness in African-American Prostate Cancer Patients. Sci. Rep. 8 (1), 16335. 10.1038/s41598-018-34637-8 30397274 PMC6218553

[B3] Altman D. G. Bland J. M. (1994). Statistics Notes: Diagnostic Tests 1: Sensitivity and Specificity. BMJ 308 (6943), 1552. 10.1136/bmj.308.6943.1552 8019315 PMC2540489

[B4] Asplund J. Kauppila J. H. Mattsson F. Lagergren J. (2018). Survival Trends in Gastric Adenocarcinoma: A Population-Based Study in Sweden. Ann. Surg. Oncol. 25 (9), 2693–2702. 10.1245/s10434-018-6627-y 29987609 PMC6097732

[B5] Assenov Y. Ramírez F. Schelhorn S.-E. Lengauer T. Albrecht M. (2007). Computing Topological Parameters of Biological Networks. Bioinformatics 24 (2), 282–284. 10.1093/bioinformatics/btm554 18006545

[B6] Boucher B. Jenna S. (2013). Genetic Interaction Networks: Better Understand to Better Predict. Front. Genet. 4, 290. 10.3389/fgene.2013.00290 24381582 PMC3865423

[B7] Byvatov E. Schneider G. (2003). Support Vector Machine Applications in Bioinformatics. Appl. Bioinforma. 2 (2), 67–77. 15130823

[B8] Cao D. Zhao D. Jia Z. Su T. Zhang Y. Wu Y. (2020). Reactivation of Atp4a Concomitant with Intragenic DNA Demethylation for Cancer Inhibition in a Gastric Cancer Model. Life Sci. 242, 117214. 10.1016/j.lfs.2019.117214 31884095

[B9] Carvalho B. S. Irizarry R. A. (2010). A Framework for Oligonucleotide Microarray Preprocessing. Bioinformatics 26 (19), 2363–2367. 10.1093/bioinformatics/btq431 20688976 PMC2944196

[B10] Chapelle O. Scholkopf B. Zien, Eds. A. (2009). Semi-Supervised Learning (Chapelle, O. et al., Eds.; 2006) [Book reviews]. IEEE Trans. Neural Netw. 20 (3), 542. 10.1109/tnn.2009.2015974

[B11] Chawla N. V. Bowyer K. W. Hall L. O. Kegelmeyer W. P. (2002). SMOTE: Synthetic Minority Over-Sampling Technique. J. Artif. Intell. Res. 16, 321–357. 10.1613/jair.953

[B12] Chen T. Guestrin C. (2016). “XGBoost: A Scalable Tree Boosting System,” in Proceedings of the 22nd ACM SIGKDD International Conference on Knowledge Discovery and Data Mining, San Francisco, CA, August 13–17, 2016 (San Francisco, CA: Association for Computing Machinery).

[B13] Chen X. Ishwaran H. (2012). Random Forests for Genomic Data Analysis. Genomics 99 (6), 323–329. 10.1016/j.ygeno.2012.04.003 22546560 PMC3387489

[B14] Chen X. Wang M. Zhang H. (2011). The Use of Classification Trees for Bioinformatics. WIREs Data Min. Knowl. Discov. 1 (1), 55–63. 10.1002/widm.14 PMC3329156 22523608

[B15] Chicco D. Jurman G. (2020). The Advantages of the Matthews Correlation Coefficient (MCC) over F1 Score and Accuracy in Binary Classification Evaluation. BMC Genomics 21 (1), 6. 10.1186/s12864-019-6413-7 31898477 PMC6941312

[B16] Colaprico A. Silva T. C. Olsen C. Garofano L. Cava C. Garolini D. (2016). TCGAbiolinks: an R/Bioconductor Package for Integrative Analysis of TCGA Data. Nucleic Acids Res. 44 (8), e71. 10.1093/nar/gkv1507 26704973 PMC4856967

[B17] Dai W. Feng J. Hu X. Chen Y. Gu Q. Gong W. (2021a). SLC7A7 Is a Prognostic Biomarker Correlated with Immune Infiltrates in Non-small Cell Lung Cancer. Cancer Cell Int. 21 (1), 106. 10.1186/s12935-021-01781-7 33632211 PMC7905560

[B18] Dai Y. Yang G. Yang L. Jiang L. Zheng G. Pan S. (2021b). Expression of FOXA1 Gene Regulates the Proliferation and Invasion of Human Gastric Cancer Cells. Cell Mol. Biol. (Noisy-le-grand) 67 (2), 161–165. 10.14715/cmb/2021.67.2.25 34817322

[B19] Farhadian M. Rafat S. A. Panahi B. Mayack C. (2021). Weighted Gene Co-Expression Network Analysis Identifies Modules and Functionally Enriched Pathways in the Lactation Process. Sci. Rep. 11 (1), 2367. 10.1038/s41598-021-81888-z 33504890 PMC7840764

[B20] Fuxman Bass J. I. Diallo A. Nelson J. Soto J. M. Myers C. L. Walhout A. J. M. (2013). Using Networks to Measure Similarity between Genes: Association Index Selection. Nat. Methods 10 (12), 1169–1176. 10.1038/nmeth.2728 24296474 PMC3959882

[B21] Gao Y. Zhang S. Zhang Y. Qian J. (2020). Identification of MicroRNA-Target Gene-Transcription Factor Regulatory Networks in Colorectal Adenoma Using Microarray Expression Data. Front. Genet. 11, 463. 10.3389/fgene.2020.00463 32508878 PMC7248367

[B22] Gautier L. Cope L. Bolstad B. M. Irizarry R. A. (2004). affy--Analysis of Affymetrix GeneChip Data at the Probe Level. Bioinformatics 20 (3), 307–315. 10.1093/bioinformatics/btg405 14960456

[B23] Gu Q. Li Z. Han J. (2011). “Generalized Fisher Score for Feature Selection,” in Proceedings of the Twenty-Seventh Conference on Uncertainty in Artificial Intelligence (Barcelona, Spain: AUAI Press).

[B24] Heidaryan E. (2018). A Note on Model Selection Based on the Percentage of Accuracy-Precision. J. Energy Resour. Technol. 141 (4), 045501. 10.1115/1.4041844

[B25] Hu H.-M. Tsai H.-J. Ku H.-Y. Lo S.-S. Shan Y.-S. Chang H.-C. (2021). Survival Outcomes of Management in Metastatic Gastric Adenocarcinoma Patients. Sci. Rep. 11 (1), 23142. 10.1038/s41598-021-02391-z 34848751 PMC8633380

[B26] Hu Z. Tang J. Wang Z. Zhang K. Zhang L. Sun Q. (2018). Deep Learning for Image-Based Cancer Detection and Diagnosis − A Survey. Pattern Recognit. 83, 134–149. 10.1016/j.patcog.2018.05.014

[B27] Huang Y. Zhu J. Li W. Zhang Z. Xiong P. Wang H. (2018). Serum microRNA Panel Excavated by Machine Learning as a Potential Biomarker for the Detection of Gastric Cancer. Oncol. Rep. 39 (3), 1338–1346. 10.3892/or.2017.6163 29286167

[B28] Janky R. S. Verfaillie A. Imrichová H. Van de Sande B. Standaert L. Christiaens V. (2014). iRegulon: From a Gene List to a Gene Regulatory Network Using Large Motif and Track Collections. PLoS Comput. Biol. 10 (7), e1003731. 10.1371/journal.pcbi.1003731 25058159 PMC4109854

[B29] Johnson W. E. Li C. Rabinovic A. (2007). Adjusting Batch Effects in Microarray Expression Data Using Empirical Bayes Methods. Biostatistics 8 (1), 118–127. 10.1093/biostatistics/kxj037 16632515

[B30] Kang M.-H. Choi H. Oshima M. Cheong J.-H. Kim S. Lee J. H. (2018). Estrogen-Related Receptor Gamma Functions as a Tumor Suppressor in Gastric Cancer. Nat. Commun. 9 (1), 1920. 10.1038/s41467-018-04244-2 29765046 PMC5954140

[B31] Kang M.-H. Eyun S.-I. Park Y.-Y. (2021). Estrogen-Related Receptor-Gamma Influences *Helicobacter P* Infection by Regulating TFF1 in Gastric Cancer. Biochem. Biophys. Res. Commun. 563, 15–22. 10.1016/j.bbrc.2021.05.076 34058470

[B32] Katai H. Ishikawa T. Ishikawa T. Akazawa K. Isobe Y. Miyashiro I. (2018). Five-Year Survival Analysis of Surgically Resected Gastric Cancer Cases in Japan: A Retrospective Analysis of More Than 100,000 Patients from the Nationwide Registry of the Japanese Gastric Cancer Association (2001-2007). Gastric Cancer 21 (1), 144–154. 10.1007/s10120-017-0716-7 28417260

[B33] Lancashire L. J. Lemetre C. Ball G. R. (2009). An Introduction to Artificial Neural Networks in Bioinformatics-Aapplication to Complex Microarray and Mass Spectrometry Datasets in Cancer Studies. Briefings Bioinforma. 10 (3), 315–329. 10.1093/bib/bbp012 19307287

[B34] Langfelder P. Horvath S. (2008). WGCNA: An R Package for Weighted Correlation Network Analysis. BMC Bioinforma. 9, 559. 10.1186/1471-2105-9-559 PMC2631488 19114008

[B35] Li C. Hou X. Yuan S. Zhang Y. Yuan W. Liu X. (2021). High Expression of TREM2 Promotes EMT via the PI3K/AKT Pathway in Gastric Cancer: Bioinformatics Analysis and Experimental Verification. J. Cancer 12 (11), 3277–3290. 10.7150/jca.55077 33976737 PMC8100818

[B36] Lin M. Pan J. Chen Q. Xu Z. Lin X. Shi C. (2018). Overexpression of FOXA1 Inhibits Cell Proliferation and EMT of Human Gastric Cancer AGS Cells. Gene 642, 145–151. 10.1016/j.gene.2017.11.023 29129808

[B37] Lin S. Lin B. Wang X. Pan Y. Xu Q. He J.-S. (2017). Silencing of ATP4B of ATPase H+/K+ Transporting Beta Subunit by Intragenic Epigenetic Alteration in Human Gastric Cancer Cells. Oncol. Res. 25 (3), 317–329. 10.3727/096504016X14734735156265 28281974 PMC7840950

[B38] Liu J. Feng W. Liu M. Rao H. Li X. Teng Y. (2021). Stomach-Specific C-Myc Overexpression Drives Gastric Adenoma in Mice via AKT/mTOR Signaling. Bosn J Basic Med Sci 21 (4), 434–446. 10.17305/bjbms.2020.4978 33259779 PMC8292868

[B39] Lonsdale J. Thomas J. Salvatore M. Phillips R. Lo E. Shad S. (2013). The Genotype-Tissue Expression (GTEx) Project. Nat. Genet. 45 (6), 580–585. 10.1038/ng.2653 23715323 PMC4010069

[B40] Lozano-Pope I. Sharma A. Matthias M. Doran K. S. Obonyo M. (2017). Effect of Myeloid Differentiation Primary Response Gene 88 on Expression Profiles of Genes during the Development and Progression of Helicobacter-Induced Gastric Cancer. BMC cancer 17 (1), 133. 10.1186/s12885-017-3114-y 28201999 PMC5310019

[B41] Peng Z. Guan Q. Luo J. Deng W. Liu J. Yan R. (2020). Sophoridine Exerts Tumor-Suppressive Activities via Promoting ESRRG-Mediated β-Catenin Degradation in Gastric Cancer. BMC Cancer 20 (1), 582. 10.1186/s12885-020-07067-x 32571331 PMC7310191

[B42] Rana H. K. Akhtar M. R. Islam M. B. Ahmed M. B. Lió P. Huq F. (2020). Machine Learning and Bioinformatics Models to Identify Pathways that Mediate Influences of Welding Fumes on Cancer Progression. Sci. Rep. 10 (1), 2795. 10.1038/s41598-020-57916-9 32066756 PMC7026442

[B43] Rolig A. S. Shanks J. Carter J. E. Ottemann K. M. (2012). *Helicobacter P* Requires TlpD-Driven Chemotaxis to Proliferate in the Antrum. Infect. Immun. 80 (10), 3713–3720. 10.1128/IAI.00407-12 22802346 PMC3457577

[B44] Royston P. Parmar M. K. B. Sylvester R. (2004). Construction and Validation of a Prognostic Model across Several Studies, with an Application in Superficial Bladder Cancer. Stat. Med. 23 (6), 907–926. 10.1002/sim.1691 15027080

[B45] Saragoni L. Morgagni P. Gardini A. Marfisi C. Vittimberga G. Garcea D. (2013). Early Gastric Cancer: Diagnosis, Staging, and Clinical Impact. Evaluation of 530 Patients. New Elements for an Updated Definition and Classification. Gastric Cancer 16 (4), 549–554. 10.1007/s10120-013-0233-2 23423491

[B46] Shannon P. Markiel A. Ozier O. Baliga N. S. Wang J. T. Ramage D. (2003). Cytoscape: A Software Environment for Integrated Models of Biomolecular Interaction Networks. Genome Res. 13 (11), 2498–2504. 10.1101/gr.1239303 14597658 PMC403769

[B47] Subramanian I. Verma S. Kumar S. Jere A. Anamika K. (2020). Multi-Omics Data Integration, Interpretation, and its Application. Bioinform Biol. Insights 14, 1177932219899051. 10.1177/1177932219899051 32076369 PMC7003173

[B48] Sung H. Ferlay J. Siegel R. L. Laversanne M. Soerjomataram I. Jemal A. (2021). Global Cancer Statistics 2020: GLOBOCAN Estimates of Incidence and Mortality Worldwide for 36 Cancers in 185 Countries. CA A Cancer J. Clin. 71 (3), 209–249. 10.3322/caac.21660 33538338

[B49] Tang Z. Kang B. Li C. Chen T. Zhang Z. (2019). GEPIA2: An Enhanced Web Server for Large-Scale Expression Profiling and Interactive Analysis. Nucleic Acids Res. 47 (W1), W556–W560. 10.1093/nar/gkz430 31114875 PMC6602440

[B50] Thul P. J. Lindskog C. (2018). The Human Protein Atlas: A Spatial Map of the Human Proteome. Protein Sci. 27 (1), 233–244. 10.1002/pro.3307 28940711 PMC5734309

[B51] Van Cutsem E. Sagaert X. Topal B. Haustermans K. Prenen H. (2016). Gastric Cancer. Lancet 388 (10060), 2654–2664. 10.1016/S0140-6736(16)30354-3 27156933

[B52] Xiang P. Liu D. Guan D. Du Z. Hao Y. Yan W. (2021). Identification of Key Genes in Benign Prostatic Hyperplasia Using Bioinformatics Analysis. World J. Urol. 39 (9), 3509–3516. 10.1007/s00345-021-03625-5 33564912

[B53] Yang A. Zhang W. Wang J. Yang K. Han Y. Zhang L. (2020). Review on the Application of Machine Learning Algorithms in the Sequence Data Mining of DNA. Front. Bioeng. Biotechnol. 8, 1032. 10.3389/fbioe.2020.01032 33015010 PMC7498545

[B54] Yang H. Tian W. Zhou B. (2022). Sarcopenia and a 5-mRNA Risk Module as a Combined Factor to Predict Prognosis for Patients with Stomach Adenocarcinoma. Genomics 114 (1), 361–377. 10.1016/j.ygeno.2021.12.011 34933074

[B55] Ye W. Luo C. Liu F. Liu Z. Chen F. (2021). CD96 Correlates with Immune Infiltration and Impacts Patient Prognosis: A Pan-Cancer Analysis. Front. Oncol. 11, 634617. 10.3389/fonc.2021.634617 33680972 PMC7935557

[B56] Zeng T. Sun S.-Y. Wang Y. Zhu H. Chen L. (2013). Network Biomarkers Reveal Dysfunctional Gene Regulations during Disease Progression. FEBS J. 280 (22), 5682–5695. 10.1111/febs.12536 24107168

[B57] Zhang G. Liu X. Sun Z. Feng X. Wang H. Hao J. (2022). A2M Is a Potential Core Gene in Intrahepatic Cholangiocarcinoma. BMC Cancer 22 (1), 5. 10.1186/s12885-021-09070-2 34979994 PMC8722218

[B58] Zhang G. Xue Z. Yan C. Wang J. Luo H. (2021). A Novel Biomarker Identification Approach for Gastric Cancer Using Gene Expression and DNA Methylation Dataset. Front. Genet. 12, 644378. 10.3389/fgene.2021.644378 33868380 PMC8044773

[B59] Zhang Z. (2016). Introduction to Machine Learning: K-Nearest Neighbors. Ann. Transl. Med. 4 (11), 218. 10.21037/atm.2016.03.37 27386492 PMC4916348

[B60] Zhou D. Bousquet O. Lal T. Weston J. Schölkopf B. (2003). Learning with Local and Global Consistency. Adv. Neural Inf. Process. Syst. 16, 321–328.

