# Supplementary material for: ESRRG, ATP4A, and ATP4B as Diagnostic Biomarkers for Gastric Cancer: A Bioinformatic Analysis Based on Machine Learning
Source: Front Physiol. 2022 Jun 23;13:905523. doi: 10.3389/fphys.2022.905523 (PMC9262247; doi:10.3389/fphys.2022.905523)
Supplement: Supplementary file 2 [file DataSheet2.ZIP › Supplementary Tables/Supplementary Table I.docx]

**Table I** Summary of 6 microarray datasets used in this study

| **Accession** | **Platform full name** | **Platform**  **abbreviation** | **Samples**  **(tumor/normal)** | **Database**  **source** | **Region** | **Role in this study** |
| --- | --- | --- | --- | --- | --- | --- |
| GSE66229 | [HG-U133_Plus_2] Affymetrix Human  Genome U133 Plus 2.0 Array | GPL570 | 196(98/98) | GEO | ASIA | WGCNA;  Feature gene selection;  Machine learning model  Development/ Internal Validation |
| GSE19826 | [HG-U133_Plus_2] Affymetrix Human Genome U133 Plus 2.0 Array | GPL570 | 24(12/12) | GEO | CHINA | Feature gene selection;  Machine learning model  Development/ Internal Validation |
| GSE29272 | [HG-U133A] Affymetrix Human Genome U133A Array | GPL96 | 268(134/134) | GEO | CHINA | Feature gene selection;  Machine learning model  Development/ Internal Validation |
| GSE27342 | [HuEx-1_0-st] Affymetrix Human Exon 1.0 ST Array [transcript (gene) version] | GPL5175 | 160(80/80) | GEO | CHINA | Feature gene selection;  Machine learning model  Development/ Internal Validation |
| GSE54129 | [HG-U133_Plus_2] Affymetrix Human Genome U133 Plus 2.0 Array | GPL570 | 132(111/21) | GEO | CHINA | Feature gene selection;  Machine learning model  Development/ Internal Validation |
| GSE33335 | [HuEx-1_0-st] Affymetrix Human Exon 1.0 ST Array [transcript (gene) version] | GPL5175 | 50(25/25) | GEO | CHINA | Machine learning external Validation |

Note: The raw data of GSE66229 and GSE19826 has 400 and 27 samples respectively, but only normal and cancer samples from the same individuals were calculated in our research, so the samples used in this research in GSE66229 and GSE19826 were 196 and 24.

1
