# Supplementary material for: ESRRG, ATP4A, and ATP4B as Diagnostic Biomarkers for Gastric Cancer: A Bioinformatic Analysis Based on Machine Learning
Source: Front Physiol. 2022 Jun 23;13:905523. doi: 10.3389/fphys.2022.905523 (PMC9262247; doi:10.3389/fphys.2022.905523)
Supplement: Supplementary file 2 [file DataSheet2.ZIP › Supplementary Tables/Supplementary Table I.pdf]

Table I Summary of 6 microarray datasets used in this study

Accession	Platform full name	Platform abbreviation	Samples (tumor/normal)	Database source	Region	Role in this study
GSE66229	[HG-U133_Plus_2] Affymetrix Human Genome U133 Plus 2.0 Array	GPL570	196(98/98)	GEO	ASIA	WGCNA; Feature gene selection; Machine learning model Development/ Internal Validation
GSE19826	[HG-U133_Plus_2] Affymetrix Human Genome U133 Plus 2.0 Array	GPL570	24(12/12)	GEO	CHINA	Feature gene selection; Machine learning model Development/ Internal Validation
GSE29272	[HG-U133A] Affymetrix Human Genome U133A Array	GPL96	268(134/134)	GEO	CHINA	Feature gene selection; Machine learning model Development/ Internal Validation
GSE27342	[HuEx-1_0-st] Affymetrix Human Exon 1.0 ST Array [transcript (gene) version]	GPL5175	160(80/80)	GEO	CHINA	Feature gene selection; Machine learning model Development/ Internal Validation
GSE54129	[HG-U133_Plus_2] Affymetrix Human Genome U133 Plus 2.0 Array	GPL570	132(111/21)	GEO	CHINA	Feature gene selection; Machine learning model Development/ Internal Validation
GSE33335	[HuEx-1_0-st] Affymetrix Human Exon 1.0 ST Array [transcript (gene) version]	GPL5175	50(25/25)	GEO	CHINA	Machine learning external Validation

Note: The raw data of GSE66229 and GSE19826 has 400 and 27 samples respectively, but only normal and cancer samples from the same individuals were calculated in our research, so the samples used in this research in GSE66229 and GSE19826 were 196 and 24.
